# The transition from intra to extra-uterine life in late preterm infant: a single-center study

**DOI:** 10.1186/s13052-016-0293-0

**Published:** 2016-09-22

**Authors:** M. P. De Carolis, G. Pinna, C. Cocca, S. A. Rubortone, C. Romagnoli, I. Bersani, S. Salvi, A. Lanzone, S. De Carolis

**Affiliations:** 1 Department of Paediatrics, Division of Neonatology, Catholic University of Sacred Heart, Universitary Hospital A. Gemelli, Largo Gemelli 8, 00168 Rome, Italy; 2 Department of Obsterics and Gynecology, Catholic University of Sacred Heart, Rome, Italy

**Keywords:** Late preterm infant, Transition, Birth, Cardiopulmonary resuscitation, Thermoregulation

## Abstract

**Background:**

Infants born at 34 to 36 weeks of gestation (late preterm) are at greater risk for adverse outcomes than those born at 37 weeks of gestation or later. Aim of this paper is to examine risk factors for late preterm births and to investigate the complications of the transition period in late preterm infants (LPIs).

**Methods:**

All consecutive late preterm deliveries, excluded stillbirths, were included. Maternal and neonatal data, need for delivery room resuscitative procedures, temperature at birth (T1) and two hours after the admission (T2) were analyzed in all LPIs stratified by Gestational Age (GA) and divided into three groups (34, 35 and 36 weeks).

**Results:**

Two hundred seventy-six LPIs were analyzed. Pregnancy complications were present in 72 mothers (26.1 %), more frequently at 34 weeks of gestation respect to 35 and 36 weeks (*p* = 0.008, *p* = 0.006 respectively). Forty seven LPIs (17.1 %) needed for any resuscitation and 37 (13.4 %) were ventilated at birth. LPIs at 34 weeks were significantly more likely to receive ventilation respect to those at 35 and 36. At T1 the mean temperature resulted lower at 34 weeks respect to 36 weeks (*p* = 0.03). At T2 respect to T1, the rate of normothermic neonates increased at 35 and 36 weeks (*p* = 0.003, *p* = 0.005, respectively).

Hypoglicemia rate was similar among the groups; 66.7 % of hypoglicemic neonates were hypothermic at T1. The rate of respiratory diseases and NICU admission decreased with increasing GA. Higher number of neonates ventilated at birth developed respiratory disorders respect to those unventilated (40.5 % vs 8.4 %; *p* < 0.001).

**Conclusions:**

Transition period in LPIs may become critical, as resuscitation strategies can be required and heat loss can occur. LPIs, especially at 34 gestational weeks, are higher-risk group needing adequate and targeted management at birth.

## Background

The fetal-to-neonatal transition at birth is characterized by major physiological changes in respiratory and hemodynamic function and in thermoregulation [1].

Late preterm infants (LPIs), defined as neonates born between 34^0/7^ and 36^6/7^ weeks of gestation [2], are physiologically and metabolically immature at birth, and can be lacking of the self-regulatory ability to appropriately respond to the extra-uterine environment. Despite being considered as “near term”, LPIs have higher rates of morbidity and mortality respect to term neonates [3]. Specifically, hypothermia and respiratory morbidity have been found to be more common in this group compared with term neonates [4]. It is reported that LPIs may experience delayed or inadequate transition to the extra-uterine environment [5] and are considered at higher risk for developing respiratory distress [6].

The aim of the present study was to evaluate maternal risk factors for late preterm (LP) delivery and to investigate the complications during transition period in LPIs, particularly by evaluating the need for resuscitation and the risk of hypothermia.

## Methods

A retrospective study of all the consecutive LP deliveries, identified from perinatal data base of the University Hospital “A.Gemelli”, Rome, Italy, from January 1 to December 31, 2013 was conducted. Maternal and neonatal informations were collected from medical records. The approval of the Ethics Committee of our Institution was obtained. Pregnancies complicated by stillbirths were excluded.

The following maternal and obstetric factors were evaluated: age, ethnicity, parity, type of pregnancy (singleton or multiple gestations), pre-existing medical conditions, pregnancy complications, presence of labour, delivery mode and reasons for C-section [7], administration of antenatal steroid (ANCS) therapy.

The pre-existing medical conditions considered were autoimmune, cardiovascular, infectious and neurological diseases, thyroid disorders, cancer, hematological conditions or thrombosis.

Pregnancy complications included were: diabetes (gestational diabetes and diabetes types 1 and 2), hypertensive disorders (gestational hypertension and preeclampsia) [8] and intrahepatic cholestasis. Intrauterine growth restriction (IUGR) was defined as an estimated fetal weight < the 10^th^ percentile for the gestational age (GA). ANCS therapy was considered when a complete course, consisting of two doses of 12 mg betamethasone 24-h apart, was administered.

At the time of delivery, the presence of preterm premature rupture of membranes (pPROM), defined as rupture of membranes occurring before the onset of labour, spontaneous labour, defined by presence of uterine contractions leading to delivery, and placental accidents, including placental abruption and placenta praevia, were evaluated.

Neonatal variables included: GA, birth weight (BW), small for gestational age (SGA) defined as BW < the 10^th^ percentile, gender and presence of major malformations. GA was determined according to first-trimester crown–rump length.

From the delivery room (DR), informations regarding the neonatal status at birth were recorded including the resuscitative procedure carried out according to the American Academy of Pediatrics guidelines [9]. Initial ventilation was provided with the NeoPuff device (Fisher & Paykel Healthcare Inc, Irvine, CA), by mask or endotracheal tube.

Measurement of body temperature was performed, as part of routine quality assurance processes, at birth (T1) and two hours after the admission (T2). Measures to reduce the risk of hypothermia were adopted in all LPIs in DR [10]. Temperatures were classified as: hyperthermia (>37.5°C), normothermia (36.5–37.5 °C), mild hypothermia (36.0–36.4 °C), moderate hypothermia (32.0–35.9 °C), and severe hypothermia (<32.0 °C) as indicated by the WHO.

During the immediate postpartum period recovery, a close monitoring of glycemia was performed and hypoglycemia was defined as plasma glucose level <40 mg/dl [11]. The occurrence of respiratory pathologies, such as transient tachypnea of the newborn (TTN) or respiratory distress syndrome (RDS)was evaluated. Infants requiring ventilatory support were admitted to the NICU.

### Statistical analysis

LPIs were stratified by GA and divided into three groups (34, 35 and 36 weeks); 34 completed weeks included births at 34^0/7^ weeks through 34^6/7^ weeks and so on through 36 weeks.

Descriptive statistics of continuous variables were reported as mean ± standard deviation for normally distributed data, or median (range) for skewed data. Comparisons across the GA groups were performed with the Kruskal-Wallis equality-of-populations rank test or ANOVA as appropriate. Wilcoxon matched-pairs signed-ranks or Student’s *t* test were used as *post-hoc* test. Categorical variables, reported as number (percent), were analyzed using Chi-square test. For the logistic regression analysis maternal and neonatal variables with *p* < 0.05 in the univariate analysis were included. Differences were considered statistically significant for *p* < 0.05. Analyses were conducted in SPPS for Windows (Version 15.0.1).

## Results

In 2013, in our center, 14.2 % of all births were premature (475/3334). The largest contribution to these preterm births was from LP deliveries (58.1 %, 276/475): 22.4 % (*N* = 62) born at 34 weeks, 27.8 % (*N* = 77) at 35 and 49.6 % (*N* = 137) at 36 weeks. Among LPIs, twins represented 22.4 % (*N* = 62): 48 were dichorionic and 14 monochorionic twins.

Maternal, obstetric and neonatal characteristics of mother-neonate pairs are shown in Table 1 for overall cases and separately according to GA groups.

**Table 1:** Demographic characteristic of mother-neonate pairs

Characteristics		Gestational Age (Weeks^+ days of gestation^)			
	Total (*n* = 276)	34^0/7^ (*n* = 62)	35^0/7^ (*n* = 77)	36^0/7^ (*n* = 137)	P*
Mothers					
Maternal age, years *mean±SD (range)*	34±6 (16–52)	35±7 (23–44)	34±6 (23–52)	33±6 (16–46)	0.75
Immigrant mothers, n (%)	75 (27.2)	17 (27.4)	19 (24.7)	39 (28.5)	0.83
Primiparous, n (%)	97 (35.1)	22 (45.5)	29 (37.7)	46 (33.6)	0.83
Singleton gestation, n (%)	227(82.7)	40 (64.5)	63 (81.8)	124 (90.5)	0.36
Pre-existing medical conditions, n (%)	28 (10.1)	5 (8.1)	12 (15.6)	11 (8.1)	0.17
Pregnancy complications, n (%)	72 (26.1)	28 (45.2)	15 (19.5)	29 (21.6)	<0.001
Diabetes, n (%)	29 (10.5)	5 (8.1)	7 (9.1)	17 (12.4)	0.58
Preeclampsia, n (%)	13 (4.7)	9 (14.5)	2 (2.6)	2 (1.4)	<0.001
Gestational hypertension, n (%)	9 (3.2)	2 (3.2)	3 (3.9)	4 (2.9)	0.9
Intrahepatic cholestasis, n (%)	14 (5.1)	8 (12.9)	2 (2.6)	4 (2.9)	0.006
IUGR, n (%)	7 (2,5)	4 (6.4)	1 (1.3)	2 (1.5)	0.08
Antenatal Steroid, n (%)	94 (34.1)	35 (56.4)	26 (33.7)	33 (24.0)	<0.001
Delivery					
PROM, n (%)	76 (27.5)	9 (14.5)	24 (31.2)	43 (31.4)	0.03
Labour, n (%)	128 (59.1)	26 (41.9)	46 (59.7)	91 (66.4)	0.005
Vaginal delivery, n (%)	86 (31.2)	13 (20.9)	24 (31.2)	49 (35.7)	0.11
PROM & Vaginal delivery, n (%)	34 (12.3)	2 (15.4)	10 (41.6)	22 (44.9)	0.03
C-section n (%)	190 (68.8)	49 (79.1)	53 (68.8)	88 (59.1)	0.17
C-section indications					
Maternal, n (%)	36 (18.9)	11 (22.4)	14 (26.4)	11 (12.5)	0.04
Placental, n (%)	51 (26.8)	13 (26.5)	15 (28.3)	23 (26.3)	0.7
Fetal, n (%)	87 (45.7)	24 (48.9)	21 (39.6)	42 (47.7)	0.33
Obstetrician, n (%)	16 (8.4)	1 (2.0)	3 (5.6)	12 (13.6)	0.09
CS without labour of all CS, n%	113 (59.5)	36 (73.5)	31 (58.5)	46 (52,3)	0.05
Neonates					
Birth Weight, g *mean±SD (range*)	2463±458 (1180–3980)	2164±424 (1200–2750)	2383±404 (1400–3215)	2644±419 (1180–3890)	<0.001
Male, n(%)	145 (52.5)	27 (43.5)	37 (48.1)	81 (59.1)	0.08
SGA, n(%)	49 (17.7)	15 (24.2)	13 (16.9)	21 (15.3)	0.31
Malformations, n(%)	29 (10.5)	9 (14.5)	7 (9.1)	13 (9.5)	0.51

*P-value is calculated among the three gestational age groups

No statistically significant difference in maternal age, ethnicity and parity was found as well as in the number of singleton pregnancies and of pre-existing maternal medical conditions. Overall, pregnancy complications were present in 26.1 %, with higher rate at 34 weeks respect to 35 and 36 weeks (*p* = 0.008, *p* = 0.006; respectively). Diabetes was the most frequent complication with a similar rate among the groups*,* whereas preeclampsia was more frequent at 34 weeks respect to 35 and 36 weeks of gestation (*p* = 0.009, *p* < 0.001; respectively) as well as intrahepatic cholestasis (*p* = 0.01, *p* = 0.006; respectively). Higher number of neonates born at 34 weeks received ANCS therapy respect to those born at 35 and 36 weeks (*p* = 0.007, *p* < 0.001; respectively).

The pPROM occurred in 27.5 % of the total LP births, with a higher rate at 35 and 36 weeks respect to 34 weeks (*p* = 0.02, *p* = 0.01; respectively), as well as the rate of pPROM associated with vaginal delivery (*p* = 0.04, *p* = 0.01; respectively). Labour was present in 59.1 % of all deliveries and the rate resulted different among the GA groups, lower in neonates born at 34 weeks respect to those born at 35 and 36 weeks (*p* = 0.03, *p* = 0.001 respectively).

No significant difference in the number of vaginal delivery as well as in the number of C-section performed for placental or fetal indications was found among the groups. Contrariwise, higher number of C-section due to maternal indications was detected at 34 and 35 weeks respect to 36 weeks. Except for the BW, the other neonatal characteristics were similar among the groups. The number of SGA neonates was similar for the neonates born either to singleton pregnancies or to twin pregnancies among the three groups.

Forty-eight LPIs (17.4 %) needed for any resuscitation procedure (free-flow oxygen and/or ventilation) and 37 (13.4 %) for ventilation. Neonates born at 34 weeks in comparison to those at 35 and 36 weeks required more ventilation (*p* = 0.01, *p* < 0.001; respectively) and more ventilation with either mask or endotracheal tube (*p* = 0.004, *p* < 0.001; respectively). No difference was also observed between single and twin neonates. Higher number of infants born at 34 weeks showed Apgar Score <7 at 1 and 5 min of life respect to those born at 35 (*p* < 0.001 for each one) and 36 weeks (*p* < 0.001, *p* = 0.009, respectively) (Table 2).

**Table 2:** Resuscitation procedures at birth and Apgar score

		Gestational Age (Weeks^+ days of gestation^)			
	Total (*n* = 276)	34^0/7^ (*n* = 62)	35^0/7^ (*n* = 77)	36^0/7^ (*n* = 137)	P*
Any Resuscitation Procedure, n(%)	48 (17.4)	25 (40.3)	12 (15.5)	11 (8.0)	<0.001
Only free-flow Oxygen, n(%)	11 (3.9)	6 (9.6)	2 (2.6)	3 (2.1)	0.03
Ventilation	37 (14.3)	19 (30.6)	10 (12.3)	8 (5.8)	<0.001
Mask, n(%)	25 (6.7)	11 (17.7)	7 (9.0)	7 (5.1)	0.01
ET tube, n(%)	12 (3.2)	8 (12.9)	3 (3.9)	1 (0.7)	0.004
Chest compressions, n(%)	1 (0.3)	0	1(1.3)	0	0.27
Drugs, n(%)	2 (0.7)	1 (1.6)	1 (1.3)	0	0.13
Umbilical Venous Catheter, n(%)	4 (1.4)	2 (3.2)	2 (2.6)	0	0.13
Apgar score^1^					
Median (range)	9 (1–10)	8 (1–9)	9 (2–9)	9 (4–10)	<0.001
< 7, n(%)	25 (9.1)	17 (27.4)	4 (5.2)	4 (2.9)	<0.001
Apgar score^5^					
Median (range)	9 (2–10)	9 (6–10)	9 (2–10)	10 (7–10)	<0.001
< 7, n(%)	5 (1.8)	3 (4.8)	2 (2.6)	0	0.04

*P-value is calculated among the three gestational age groups

The comparison between the 37 LPIs ventilated and the 239 LPIs not undergone to this procedure is outlined in Table 3. A higher number of ventilated neonates was born at 34 weeks. The C-section rate was significantly higher instead the presence of labour was lower in ventilated neonates compared to those unventilated.

**Table 3:** Demographic variables according to the need for ventilation in Delivery Room

	Yes	No	*P*
	*N* = 37	*N* = 239	
Birth Weight, g mean±SD (range)	2316±383 (1180–2900)	2486±465 (1200–3890)	0.03
Gestational age (weeks)			<0.001
34^0/7^ to 34^6/7^, n (%)	19 (51.3)	43 (17.9)	
35^0/7^ to 35^6/7^, n (%)	10 (27.1)	67 (28.0)	
36^0/7^ to 35^6/7^, n (%)	8 (21.6)	129 (53.9)	
Male, n(%)	20 (52.6)	135 (56.7)	0.78
SGA, n (%)	4 (10.5)	45 (18.9)	0.23
Twins, n (%)	11 (28.9)	51 (21.4)	0.25
Antenatal Steroids, n (%)	19 (50.0)	79 (33.2)	0.03
Labour, n (%)	12 (31.6)	146 (61.1)	0.001
C-section, n (%)	33 (89.1)	157 (65.7)	0.004
Malformations, n (%)	7 (18.9)	22 (9.2)	0.07

Factors associated with the needing for ventilation were analyzed by multiple regression analysis (Table 4). The GA resulted the strongest factor among the all other variables: the decreasing of the GA was associated with the increasing of the need for ventilation.

**Table 4:** Multivariate model of factors associated with the need for ventilation

	Odds Ratio	95 % Confidence Interval	*P*
GA			
35 weeks (vs 34)	0.38	0.15–0.94	0.037
36 weeks (vs 34)	0.67	0.06–0.43	<0.001
Preeclampsia	0.93	0.41–2.12	0.86
Antenatal Steroids	0.96	0.44–2.11	0.29
Spontaneous Labor	0.50	0.20–1.25	0.13
C-section	2.36	0.66–8.42	0.18

**Fig. 1:**
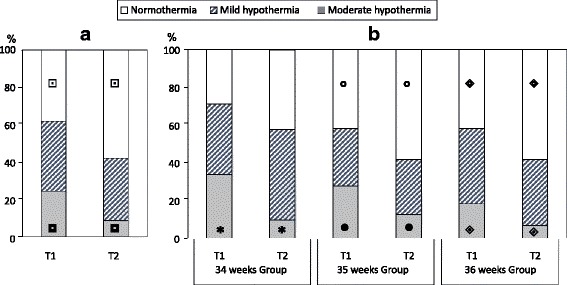
Distribution of normothermia and hypothermia in all LPIs (**a**) and in three groups of gestational age (**b**) at T1 and T2. (In all LPIs: normothermia  *p < 0.001*, moderate hypothermia  *p* < 0.001; in 34 weeks Group: moderate hypothermia _*_ *p < 0.001*; in 35 weeks Group: normothermia ○ *p = 0.003*, moderate hypothermia ● *p = 0.04;* in 36 weeks Group: normothermia  *p* = 0.005, moderate hypothermia  *p* = 0.003)

In all LPIs and in each GA group the mean temperature increased from T1 to T2 (*p* < 0.001). However, at T1, the mean temperature resulted lower in neonates of 34 respect to those of 36 weeks (*p* = 0.03) (Table 5). As shown in Fig. 1a, in all LPIs, the rate of normothermic neonates significantly increased from T1 to T2 where moderate hypothermia significantly decreased. When all the LPIs were divided according to the GA at birth, the same type of temperature changing was found in neonates of 35 and 36 weeks. Instead, at 34 weeks, only the rate of moderate hypothermia significantly decreased without a significant increase of normothermic infants (Fig. 1b). The same trend was observed in neonates born either from singleton or twin pregnancies.

**Table 5:** Temperature at admission (T1) and after two hours (T2), hypoglycemia, respiratory disorders and need of NICU admission

		Gestational Age (Weeks^+ days of gestation^)			
	Total (*n* = 276)	34^0/7^ (*n* = 62)	35^0/7^ (*n* = 77)	36^0/7^ (*n* = 137)	P*
Temperature at T1, °C *mean±DS (range)*	36.2 ± 0.6 (33.6–37.9)	36.1 ± 0.7 (33.6–37.6)	36.3 ± 0.7 (34.0–37.9)	36.3 ± 0.5 (34.7–37.3)	0.10
Temperature at T2, °C *mean±DS (range)*	36.5 ± 0.5 (35.0–37.5)	36.4 ± 0.5 (35.0–37.5)	36.4 ± 0.5 (35.0–37.4)	36.4 ± 0.4 (34.8–37.1)	0.51
Hypoglicemia, n (%)	27 (9.7)	9 (14.5)	5 (6.5)	13 (7.3)	0.28
Respiratory disorders, n (%)	34 (12.3)	18 (29.9 %)	7 (9.1)	9 (6.5)	<0.001
TTN, n (%)	22 (7.9)	10 (16.1)	4 (5.2)	8 (5.8)	0.01
RDS, n (%)	12 (4.3)	8 (12.9)	3 (3.9)	1 (0.7)	0.004
NICU admission, n (%)	14 (5.1)	8 (12.9)	5 (6.5)	1 (0.7)	0.001

*Abbreviations*: *NICU* Neonatal Intensive Care Unit, *RDS* Respiratory Distress Syndrome, *TTN* Transient Tachypnea of the Newborn

*P-value is calculated among the three gestational age groups

The hypoglicemia rate was similar among the groups, while the rate of respiratory diseases decreased with increasing GA. Sixteen of 24 (66.7 %) hypoglicemic neonates were hypothermic at T1. Fifteen of 37 (40.5 %) neonates requiring ventilation at birth and 20 of 239 (8.4 %) unventilated neonates developed respiratory disorders (*p* < 0.001). The number of neonates admitted to NICU was higher at lower GA (Table 5).

## Discussion

LPIs have gained attention over the past several years because of the annual worldwide increased prevalence of LP births representing a growing public health concern since they are at risk of short and long-term morbidity and mortality [12]. An international meta-analysis found that the prevalence of moderate and LP births ranged from 4.4 % and 10.0 % [13]. Our study confirms this data since LPIs represented the 8.2 % of all births and the 58.1 % of all preterm births.

Since 2005 when the National Institute of Child Health and Human Development (NICHD) workshop proposed the definition of LPIs [2], several trials have been carried out to evaluate their clinical issues [14–16]. However, even if events occurring in the first moments of life can have considerable short and long-term consequences, only few studies reporting clinical data about the transition period in LPIs (such as the Apgar score values) have been published [15, 17–20].

The multicenter study by de Almeida et al. focused the attention on the resuscitation procedure showing that that LPIs required more resuscitation procedures respect to term infants: however, this is only one of the aspects of the transition period [5].

In our single center study, we retrospectively evaluated maternal and perinatal factors associated with LP birth and with the need for resuscitation procedures; the incidence of hypothermia in LPIs stratified by GA was also encountered.

Regarding the risk factors for LP delivery, our results showed that pregnancy complications were more frequent at 34 than at 35 and 36 weeks of gestation. Recently, Khashu M et al. [19] found an association between LP births and pregnancy complications, in particular hypertension, diabetes and pPROM, in all LPIs respect to term neonates. It is interesting to note that our study highlights a different distribution of pregnancy complications among the groups: preeclampsia and intrahepatic cholestasis occurred more frequently at 34 weeks, while pPROM at 35–36 weeks. Potential underlying reason for this distribution can include the conservative management of pregnancy complicated by pPROM in uninfected mothers in absence of maternal or fetal contraindications [21].

If the rate of multiple gestations in our study (22.4 %) is in accordance with the data from literature, [22], the C-section rate resulted higher [23]. This high rate of C-section is mainly due to the fact that our center is a tertiary-level hospital where high-risk pregnancies are usually referred. It is also interesting to note that the highest number of pre-labour C-section due to pregnancy complications, occurred at 34 weeks.

In our institution, according to the international guidelines, ANCS therapy is offered to women at risk of preterm birth up to 34^+6^ weeks of gestation and to all women for whom an elective C-section is planned prior to 38^+6^ weeks of gestation [24]. This treatment was carried out in 34 % of all LP pregnancies, with the higher rate at 34 weeks (56.4 %) when the highest incidence of both pregnancy complications and pre-labour C-section occurred. Besides the 34^th^ gestational week, the LP births were mainly constituted of spontaneous preterm births due to preterm labour or to urgent C-section for obstetric indications: therefore, the ANCS therapy was not administered. The effectiveness of ANCS therapy in reducing the incidence of RDS in LPIs is reported [25] and a lower rate of TTN has also been recently described [26]. However, no data are available about the effects of ANCS therapy on neonatal transition period. The NICHD is currently conducting a large randomized multicentric trial that might contribute to clarify this issue since cortisol is the major regulatory hormone for neonatal adaption at birth, facilitating the neonatal respiratory adaptation [27] and promoting the adipose tissue maturation against the cold stress [28].

In the present study, the transition to the extrauterine environment among LPIs resulted more difficult at lower gestational ages, as showed by the increasing needing for resuscitation procedures at birth such as ventilation and intubation. In the multivariate analysis, in fact, the GA plays the major role to hinder respiratory transition. The increased needing of DR interventions at 34 weeks, despite the highest rate of ANCS therapy, may be related to both their higher grade of lung immaturity, which predisposes to a delay in clearing fetal lung fluid and inefficient gas exchange, but also to the higher number of pregnancy complications and pre-labour C-section.

Another important aspect of the transition period is the thermoregulation. Laptook A et al. showed an elevated risk of hypothermia either in DR and after admission in Nursery in LPIs [29]. During the transition period LPIs are predisposed to cold stress because of the greater heat loss due to their physical characteristics and since brown adipose tissue is not properly developed in terms of quantity and efficacy [30].

In our study, large fluctuations in temperature were found in all GA groups, both at T1 and T2. The presence of hypothermic neonates in all groups proved the inability to maintain body temperature within the normal range. It is interesting to note that the temperature regain at T2 is higher in infants born at 35 and 36 weeks of gestation respect to those born at 34 weeks. As well as a worse respiratory adaptation, LPIs born at 34 gestation are more likely to experience disruptions in thermoregulation. Our data showed that resuscitation and hypothermia caused an increased risk of neonatal morbidities: respiratory disorders, hypoglycemia and consequently a higher rate of NICU admission.

Although these results are limited by the relatively small sample size and by the retrospective nature of the study and they could be considered a reflection of our clinical practice, our study highlights the importance of an accurate monitoring of LPIs during the transition period.

## Conclusion

In conclusion, we would like to underline that, among LPIs, neonates born at 34 weeks are at higher risk of resuscitation and hypothermia, so that they could be considered as *moderate r*ather than *late* preterm. Consequently, in addition to the worldwide efforts to reduce preterm birth, it is mandatory to improve standards of care and interventions in LPIs, immediately following delivery.

## Abbreviations

ANCS: Antenatal steroid
BW: Birth weight
DR: Delivery room
GA: Gestational age
IUGR: Intrauterine growth restriction
LP: Late preterm
LPIs: Late preterm infants
pPROM: Preterm premature rupture of membranes
RDS: Respiratory distress syndrome
SGA: Small for gestational age
TTN: Transient tachypnea of the newborn
